# Cellular senescence and the skeleton: pathophysiology and therapeutic implications

**DOI:** 10.1172/JCI154888

**Published:** 2022-02-01

**Authors:** Sundeep Khosla, Joshua N. Farr, David G. Monroe

**Affiliations:** Kogod Center on Aging and Division of Endocrinology and Metabolism, Mayo Clinic, Rochester, Minnesota, USA.

## Abstract

Cellular senescence is a fundamental aging mechanism that is currently the focus of considerable interest as a pathway that could be targeted to ameliorate aging across multiple tissues, including the skeleton. There is now substantial evidence that senescent cells accumulate in the bone microenvironment with aging and that targeting these cells prevents age-related bone loss, at least in mice. Cellular senescence also plays important roles in mediating the skeletal fragility associated with diabetes mellitus, radiation, and chemotherapy. As such, there are ongoing efforts to develop “senolytic” drugs that kill senescent cells by targeting key survival mechanisms in these cells without affecting normal cells. Because senescent cells accumulate across tissues with aging, senolytics offer the attractive possibility of treating multiple age-related comorbidities simultaneously.

## Introduction

The past 30 years has witnessed an explosion in our understanding of bone biology that has provided insights into the pathophysiology of skeletal diseases and driven new therapeutic advances. As early as 1980 it was known that the development of bone-resorbing osteoclasts required the presence of bone marrow stromal or osteoblast-lineage cells (1). However, the precise factor(s) produced by osteoblastic cells that were required for osteoclast development remained unclear until the late 1990s, when the osteoprotegerin/receptor activator of NF-κB ligand (RANKL)/RANK system was unraveled through a combination of mouse genetic and conventional protein purification techniques (for a historical review, see ref. 2). These fundamental developments in bone biology provided a novel therapeutic target, RANKL, which was also found to be increased on multiple cell types, including osteoblast-lineage cells, in the bone marrow of postmenopausal women (3). The progression of these discoveries from cellular and mouse models to human studies culminated in 2009 in the pivotal clinical trial of denosumab, a human monoclonal antibody against RANKL, for the prevention of fractures in postmenopausal women and subsequent approval of the drug (4). More recently, the development of romosozumab, a humanized monoclonal antibody against sclerostin, followed a similar path from basic discoveries on Wnt signaling in bone to preclinical and then clinical studies, leading to eventual drug approval for clinical use (reviewed in ref. 5). Denosumab and romosozumab added to the existing arsenal of osteoporosis drugs (four bisphosphonates, raloxifene, teriparatide, and abaloparatide), ensuring a range of treatment options for patients with osteoporosis and the possibility that this potentially devastating age-related disease could be vastly reduced in scope.

Regrettably, this prospect has not proven to be the case, and currently the majority of older patients with osteoporosis remain untreated. For example, only 15% of patients received appropriate treatment following a hip fracture (e.g., a bisphosphonate) in 2004, and this figure decreased markedly, to 3%, in 2013 (6), despite the availability of additional treatment options; unfortunately, this dismal rate of appropriate treatment for secondary fracture prevention has not improved in the intervening years (7). This “crisis” in osteoporosis treatment has several underlying causes (7), some specific to osteoporosis and others related to issues common to multiple diseases associated with aging (cardiovascular disease, diabetes, osteoarthritis, and others). The osteoporosis-specific causes include largely misplaced concerns among patients and some physicians regarding rare bisphosphonate-related side effects such as osteonecrosis of the jaw and atypical femur fractures. These concerns generally do not consider the tremendous benefits of these drugs, when used appropriately, for fracture prevention relative to the risk of these rare side effects (7). Additional causes of the lack of appropriate treatment of osteoporosis include poor coordination of health care systems for fracture prevention as well as inadequate access to appropriate diagnosis and treatment (7).

Relevant to this discussion, however, is the broader concept of treating each disease of aging separately versus recognizing, as championed by the “geroscience hypothesis,” that manipulation of fundamental aging mechanisms may delay (in parallel) the appearance or severity of multiple chronic diseases because these diseases share the same underlying risk factor — namely, aging (8). Although unproven as yet in rigorous human clinical trials, there is now substantial preclinical evidence in support of this hypothesis. Moreover, the possibility that drugs targeting fundamental aging mechanisms may complement the growing arsenal of osteoporosis-specific drugs provides some hope that we may be able to address growing gaps in osteoporosis treatment by including it as part of a broader approach to address multiple diseases of aging.

## Overview of fundamental aging mechanisms

**Figure 1:**
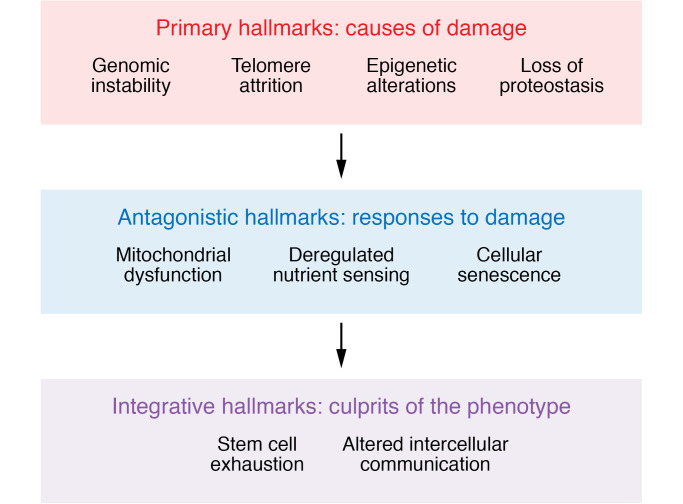
Hallmarks of aging grouped by whether they are primary causes of damage; compensatory or antagonistic responses to it; or the end result (integrative) of the previous two hallmarks. Adapted with permission from *Cell* (9).

In a landmark paper, López-Otín and colleagues proposed nine fundamental hallmarks of aging, which shared three characteristics: (a) they manifested during natural chronological aging; (b) their experimental exacerbation accelerated aging; and (c) their experimental amelioration improved healthspan and delayed aging (9). As shown in Figure 1, these nine hallmarks could be further categorized as primary hallmarks, i.e., causes of age-associated cellular damage (genomic instability, telomere attrition, epigenetic alterations, loss of proteostasis); antagonistic hallmarks, those that represented compensatory responses to age-associated cellular damage that initially mitigated the damage but then themselves caused tissue damage when they persisted over time (deregulated nutrient sensing, mitochondrial dysfunction, cellular senescence); and integrative hallmarks, which were the end result of the primary and antagonistic hallmarks and ultimately led to the functional decline associated with aging (stem cell exhaustion, altered intercellular communication). Table 1 (adapted from Farr and Almeida, ref. 10) provides a summary, with specific examples, of experimental evidence largely from mouse models of the demonstrated role of each of these aging hallmarks in contributing to skeletal aging. These hallmarks provide a useful framework to study fundamental aging mechanisms across tissues, including bone, but it should be kept in mind that these mechanisms are highly interconnected, and systems biology approaches need to be developed to fully understand the interactions between these processes in any given tissue. Importantly, cellular senescence clearly is a result of multiple other hallmarks, including DNA damage, mitochondrial dysfunction, telomere attrition, epigenetic alterations, etc., and has been the focus of considerable interest as a pathway that could be targeted to ameliorate aging across multiple tissues, including the skeleton.

## Cellular senescence as a key aging mechanism

**Figure 2:**
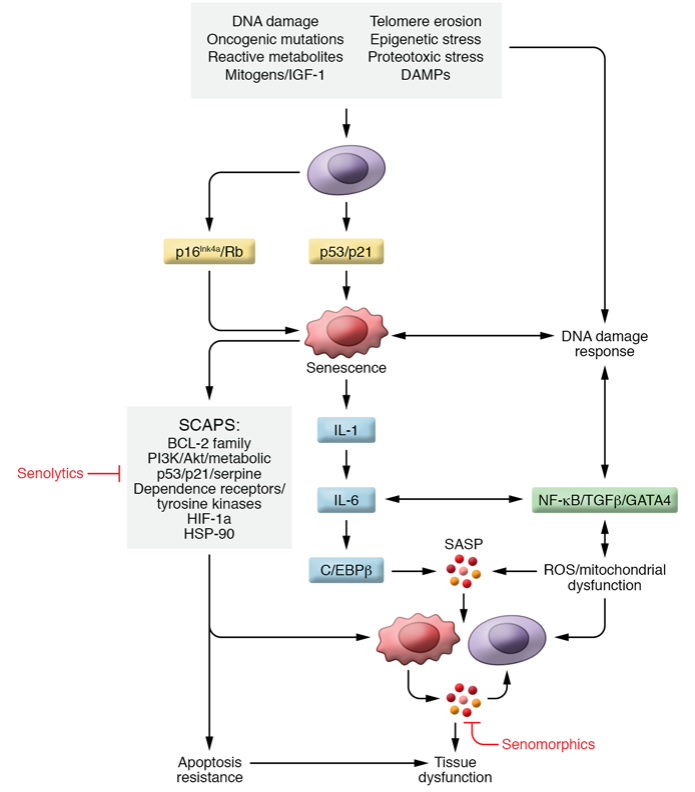
Working model of the senescence pathway based on a large number of in vitro and animal studies. The top box lists cellular stressors that trigger activation of key senescence inducers (p16^Ink4a^/Rb and p21^Cip1^/p53). This leads to activation of senescence mediators, which, in turn, promote the secretion of the proinflammatory SASP, resulting in tissue dysfunction. Senescent cells also activate senescent cell antiapoptotic pathways (SCAPs) that are the target of “senolytic” drugs. By contrast, “senomorphic” drugs do not kill senescent cells but rather inhibit the production and/or secretion of the SASP.

Although cellular senescence was originally described by Hayflick in the context of replicative senescence of primary human fibroblasts in culture (11), our understanding of this fundamental mechanism of aging has expanded greatly, and it is now well established that cellular senescence occurs not only in vitro but also in vivo, and not just in proliferating but also in terminally differentiated, nondividing cells (e.g., osteocytes and neurons) (12). Phenotypically, cellular senescence is characterized by growth arrest, altered chromatin, increased metabolic activity, and resistance to apoptosis (8). Senescent cells also develop the senescence-associated secretory phenotype (SASP), consisting of proinflammatory cytokines, chemokines, and extracellular matrix–degrading (ECM-degrading) proteins, which have deleterious paracrine and systemic effects (13–17). Figure 2 provides a working model of the senescence pathways based on a large number of in vitro and animal studies (12). Briefly, various cellular stressors (DNA damage, telomere erosion, and others) trigger activation of the key senescence inducers, including the p16^Ink4a^/Rb and p21^Cip1^/p53 pathways. These inducers subsequently lead to activation of a number of senescence mediators, including NF-κB, TGF-β, GATA4, IL-1, IL-6, and C/EBP-β, which, in turn, promote the secretion of the proinflammatory SASP, leading ultimately to tissue dysfunction. Also depicted in Figure 2 is the activation of senescent cell antiapoptotic pathways (SCAPs) that confer the resistance to apoptosis characteristic of senescent cells, but also make them vulnerable to a new class of drugs — “senolytics,” which target SCAPs and lead to apoptosis of senescent cells without harming healthy cells (18). In addition, Figure 2 shows the site of action of “senomorphic” drugs that do not kill senescent cells but rather inhibit the secretion of the SASP, including existing compounds (e.g., JAK/STAT pathway inhibitors, mTOR inhibitors) as well as potential future compounds that may inhibit the SASP through novel mechanisms (13).

In addition to the senescence pathways depicted in Figure 2, senescent cells have several additional characteristics that are the focus of ongoing studies. Specifically, senescent cells appear to evade immune clearance, and this likely contributes to their accumulation with aging (8); however, the underlying mechanisms of this immune evasion and whether they involve known “don’t eat me” signals such as CD47-SIRPα (19, 20) remain unclear. In addition, the interactions between senescent cells and the aging ECM require further study. It is known, for example, that senescent cells secrete factors that modify and remodel the ECM (e.g., matrix metalloproteinases [MMPs]), and conversely, the aging ECM likely influences senescent cell accumulation and survival (reviewed in refs. 21, 22). However, little is known at present regarding possible interactions between the ECM and senescent cells in the skeleton.

As the number of studies related to cellular senescence has expanded, it is important to enumerate consistent, broadly accepted criteria for defining a senescent cell. For example, some studies have only used staining for senescence-associated β-galactosidase (SA-β-gal), which is linked to increased lysosomal mass in senescent cells, as a senescence marker; however, SA-β-gal is neither required nor is it a determinant of the senescent phenotype (23). To address this issue, the International Cell Senescence Association (ICSA) has published a set of criteria and an algorithm for the definitive identification of senescent cells, particularly in vivo (24). These include initial screening of tissue samples for SA-β-gal or lipofuscin accumulation, which is also associated with lysosomal malfunction in senescent cells (24). Further verification involves demonstrating increased expression of the cell cycle genes *p16^Ink4a^* and *p21^Cip1^* and decreases in proliferation markers as well as in lamin B1 (*LMNB1*), a major component of the nuclear lamina that is downregulated in senescent cells (24). Finally, senescence can be established by demonstration of increased SASP markers, and perhaps most specific is the identification of telomeric DNA damage using assays such as the senescence-associated distension of satellites (SADS) (25) or telomere-associated foci (TAFs) (24, 26). As detailed below, it is possible that some of the discrepancies in the literature related to senescence in skeletal cells may be explained by varying and, in some cases, incomplete approaches to defining cellular senescence in vivo.

## Role of cellular senescence in age-related bone loss

### Senescent cells in the bone microenvironment.

Using the Hayflick method to evaluate senescence in vitro following serial passaging (11), Stenderup et al. (27) demonstrated that bone marrow stromal cells (MSCs) from aged human donors exhibited an increased number of SA-β-gal^+^ cells per population doubling as compared with MSCs from young donors. In order to definitively establish the presence of senescent cells in vivo in the bone microenvironment, our group isolated highly enriched populations of osteoblast-lineage cells (osteoblast progenitors, osteoblasts, and osteocytes) as well as bone marrow hematopoietic cells (B and T cells, myeloid cells) from young (6 months) versus old (24 months) mice and demonstrated increased *p16^Ink4a^* mRNA expression in all of the cell types evaluated; by contrast, *p21^Cip1^* was increased primarily in the osteocytes (28). Using a more specific assay, we also found an increase in the percentage of osteocytes harboring SADS in bones from the old compared with the young mice (28). In parallel human studies, *p16^Ink4a^* and *p21^Cip1^* mRNA expression by quantitative reverse transcriptase PCR was significantly elevated in bone biopsy samples from elderly compared with young women. Corroborating these findings, 23 of 36 SASP genes analyzed were upregulated in the osteocyte-enriched fractions from the old compared with the young mice; interestingly, 26 of the 36 SASP factors were also highly upregulated in the myeloid cells isolated from the old versus young mice (28).

These studies demonstrated the presence of senescence with aging in mice and in humans in multiple cell types in the bone microenvironment, which included not just skeletal cells, but also immune cells. Indeed, given the proximity of immune cells in the bone marrow to skeletal cells, it is perhaps important to put our findings in the broader context of age-related inflammation and/or senescence of immune cells. Thus, the concept of “immunosenescence” is well established in the immunology literature; however, the relationship of this concept, which is broadly defined as a “multifaceted and dynamic pattern of changes within the immune system during aging, leading to defects in both adaptive and innate immunity” (29), versus cellular senescence of immune cells, which is increasingly defined using very specific criteria (e.g., criteria developed by ICSA) (24), remains unclear. The immune cell population that has perhaps been most extensively studied and defined as becoming senescent with aging are T cells (reviewed in ref. 29). This has been best characterized for CD8^+^ cytotoxic T cells, which become senescent with aging, and these senescent CD8^+^ T cells share similar functional changes with other senescent somatic cells, including the expression of cell cycle inhibitors, resistance to apoptosis, shortened telomeres, and secretion of a SASP (29–31). In addition, detailed profiling of aging immune cells in multiple tissues by Mogilenko et al. (32) identified a subpopulation of age-associated granzyme K–expressing (GZMK-expressing) CD8^+^ T cells that contribute to “inflammaging.” Although this study did not specifically define these GZMK-expressing CD8^+^ T cells as senescent, the authors postulated that GZMK, which is a proinflammatory marker of the granzyme family, induces secretion of a proinflammatory SASP by senescent stromal cells, indicating potential crosstalk between inflammatory immune cells and senescent mesenchymal cells that amplifies the SASP of the senescent cells.

In contrast to T cells, less is known about senescence of B cells with aging, although, similarly to our findings in mice noted above, expression levels of both *p16^Ink4a^* and its related transcript *p14/p19^ARF^* increase with age in humans, particularly in pro-B, pre-B, and IgM^+^ mature B cells (33, 34). In addition, as was found in our study (28), cells of the myeloid lineage, particularly macrophages, express *p16^Ink4a^*, and there is evidence that *p16^Ink4a^*-induced cellular senescence in macrophages skews these cells toward the inflammatory M1 phenotype (35). Moreover, in recent studies, the Passos laboratory has demonstrated a novel role for neutrophils in inducing senescence (36). In these studies, neutrophils were shown to cause telomere dysfunction in mesenchymal cells in a ROS-dependent manner, and it has previously been shown that aging is associated with increased constitutive ROS production by neutrophils (37). In addition, consistent with the concept of crosstalk between inflammatory immune cells and senescent cells, senescent hepatocytes were shown to mediate the recruitment of neutrophils to the aged liver, thereby potentially spreading senescence to surrounding cells. Collectively, these findings indicate that senescence in the bone microenvironment may involve not just skeletal cells, but also inflammatory and/or senescent immune cells, and that there likely is important crosstalk between these populations. Further support for this concept comes from recent work by the Niedernhofer and Robbins laboratories demonstrating that accelerated senescence only of immune cells using immune cell–specific induction of a DNA repair defect (*Ercc1^–/fl^* mice crossed with *Vav-iCre* mice) leads to premature aging of multiple nonlymphoid tissues (e.g., vasculature, lung, liver) (38), although the skeleton was not evaluated in these studies and will be the subject of future work.

In terms of senescence of skeletal cells, findings generally consistent with ours were reported by Piemontese et al. (39), who showed higher levels of the DNA damage marker phospho–H2A histone family member X (γ-H2AX) in extracts of femoral cortical bone in old compared with young (7-month-old) mice. Moreover, the osteocytic DNA damage was also associated with increased transcript levels of *p16^Ink4a^* as well as increased protein levels of GATA4 and decreased levels of the autophagosomal cargo protein p62, both of which are senescence responses to DNA damage. Additional studies from the same group also demonstrated that, consistent with the findings from our group (28), not only osteocytes but also osteoblast progenitors developed markers of cellular senescence with aging (40).

### Interventional studies in mice.

In order to establish causality, our group has performed several interventional studies in aged mice using both senolytic and senomorphic approaches. The genetic senolytic approach involved use of the *INK-ATTAC* (INK-linked apoptosis through targeted activation of caspase) mouse model, in which either vehicle or a synthetic drug (AP20187), with no known off-target effects, is administered to transgenic mice expressing a “suicide” transgene that results in inducible elimination of *p16^Ink4a^*-expressing senescent cells without affecting non-senescent cells (41). Note that in this model, all senescent cells are targeted, so although in the AP20187-treated mice we demonstrated significant reductions in senescent (SADS^+^) osteocytes, multiple other senescent cell types in the bone microenvironment (and systemically) were also eliminated. Importantly, genetic clearance of senescent cells in 20-month-old *INK-ATTAC* mice treated for 4 months resulted in improved trabecular as well as cortical bone parameters in the AP20187- versus vehicle-treated mice (42). These changes were accompanied by reductions in osteoclast numbers and an increase in osteoblast numbers and bone formation rates on endocortical surfaces. Similar findings were noted in trabecular bone, although in this bone compartment, formation rates did not increase, but were not suppressed as would be expected following reduced bone resorption due to osteoclast-osteoblast coupling (43). Interestingly, the increase in osteoblast numbers was accompanied by a reduction in bone marrow adipocyte number, perimeter, and volume, consistent with effects of senescent cell clearance on the commitment of MSCs to the osteoblast versus the adipocyte lineage (42). Similar findings regarding effects of senescent cell clearance on MSC commitment were subsequently reported by He et al. (44), who treated old mice with two different senolytic compounds targeting Bcl-xl (navitoclax and PZ15227), thereby reducing senescent cell burden in vivo. Consistent with our findings, cultured MSCs from mice treated with either compound showed increased mineralization and decreased adipocyte formation as compared with vehicle-treated mice.

We complemented the genetic senolytic approach with pharmacological clearance of senescent cells with the senolytic cocktail of dasatinib and quercetin (hereafter referred to as D+Q) (18) as well as a senomorphic approach using the JAK inhibitor ruxolitinib, which is known to inhibit the secretion of the SASP (13). Both approaches led to skeletal effects in old mice that were virtually identical to the effects described above for genetic clearance of senescent cells. Additional control studies demonstrating the specificity of our interventions for aging included treatment of young *INK-ATTAC* mice with AP20187 or young wild-type mice with the JAK inhibitor, neither of which had any skeletal effects, as would be expected in young mice with a low burden of senescent cells (42).

**Figure 3:**
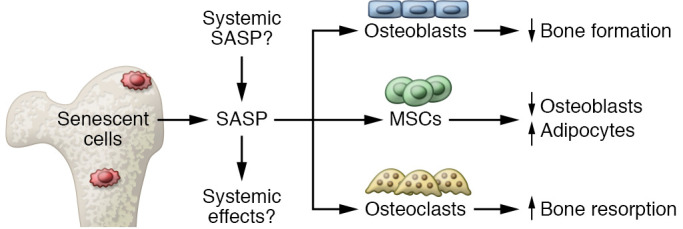
Working model of our current understanding of the contribution of cellular senescence to skeletal aging. Senescent cells accumulate in the bone microenvironment with aging and secrete a proinflammatory SASP. The contribution of the SASP from non-skeletal sites as well as the possible systemic effects of the SASP from the bone microenvironment in modulating non-skeletal aging remains unclear. The increased proinflammatory SASP in the bone microenvironment acts on osteoblasts to impair bone formation, on osteoclasts to increase bone resorption, and on MSCs to skew their lineage commitment toward adipocytes and away from osteoblasts, consistent with skeletal aging across species.

Based on these studies, Figure 3 summarizes a working model of our current understanding of the contribution of cellular senescence to skeletal aging. An important disclaimer is that not all aspects of this model have been definitively established, but rather the existing data are consistent with this model, which nonetheless provides a useful framework for future studies. In addition, considerable work needs to be done in order to define the specific components of the SASP that mediate the effects of senescent cells on osteoblasts, osteoclasts, and MSCs. With this caveat, the working hypothesis is that senescent cells accumulate in the bone microenvironment with aging and secrete a proinflammatory SASP. These senescent cells likely include osteocytes, osteoblasts, MSCs, and, as discussed above, immune cells and perhaps also endothelial and adipocytic cells. As depicted in Figure 3, the contribution of the SASP from non-skeletal sites (e.g., peripheral fat depots, which harbor markedly increased numbers of senescent cells with aging and in the setting of obesity; ref. 12) to skeletal aging, as well as the possible systemic effects of the SASP from the bone microenvironment in modulating non-skeletal aging (e.g., effects on muscle function, frailty, energy homeostasis), remains unclear and is an important area for further investigation. Nonetheless, regardless of the specific cells within or outside the skeleton contributing the SASP, the increased proinflammatory SASP in the bone microenvironment acts on osteoblasts to impair bone formation, on osteoclasts to increase bone resorption, and on MSCs to skew their lineage commitment toward adipocytes and away from osteoblasts, all of which are precisely the characteristics of skeletal aging across species (45).

The issue of not only local but also systemic effects of the SASP was addressed recently by Xu et al. (46), who demonstrated that transplantation of even small numbers of senescent cells (as few as 500,000, which would represent 0.01%–0.03% of all cells in the mouse) into the peritoneal cavity of young mice was sufficient to induce markers of frailty (e.g., reduced grip strength) in these mice. Importantly, even though the senescent cells were confined to the peritoneal cavity, markers of cellular senescence (SA-β-gal^+^ and TAF^+^ cells and *p16^Ink4a^* expression) were increased in distant tissues, such as adipose depots and skeletal muscle. Further studies evaluating the effects of transplanted senescent cells on the skeleton are ongoing, but these data do demonstrate that not only the SASP produced by cells in the bone microenvironment but potentially also the systemic SASP from other cell types may accelerate skeletal aging. An additional component of the studies by Xu et al. (46) relevant to fracture risk was the demonstration that both genetic and pharmacological (using D+Q) clearance of senescent cells improved markers of physical function (maximal walking speed, hanging endurance, grip strength, treadmill endurance, and daily activity) in old mice. Thus, an added benefit of targeting senescent cells clinically is that this approach may not only have skeletal benefits, but may also alleviate frailty and decrease the risk of falls, thereby leading to a more substantial reduction in fracture risk as compared with the skeletal-specific drugs that are in current use.

## Cellular senescence in other skeletal conditions

### Estrogen deficiency.

In addition to aging, postmenopausal estrogen deficiency represents the other major cause of bone loss (47), but evidence regarding the role of cellular senescence in estrogen deficiency–induced bone loss is conflicting; in part, this may be due to the problem noted earlier of defining senescence with a series of markers, rather than relying on a single marker (e.g., SA-β-gal). This problem is compounded by the fact that estrogen deficiency is also associated with a proinflammatory state (47) that may mimic properties of a SASP without truly reflecting underlying cell senescence.

Our group was unable to detect changes in mRNA levels of mediators of cellular senescence, including *p16^Ink4a^*, *p21^Cip1^*, or *p53*, or in the SASP panel used in our aging studies (28) in osteocyte-enriched bone samples from mice 2 months after ovariectomy (or orchiectomy) as compared with sham-operated mice (48). Similarly, 3 weeks of transdermal estrogen treatment of postmenopausal women failed to result in changes in expression of senescence or SASP genes in needle bone biopsy samples from these women. More definitively, and in contrast to our aging models, AP20187 treatment in young (6-month-old) female *INK-ATTAC* mice failed to prevent ovariectomy-induced bone loss at either trabecular or cortical sites or alter the percentage of senescent (SADS^+^) osteocytes in bone.

In contrast to our negative findings, several other groups have suggested that estrogen deficiency is associated with increased cellular senescence in bone or in osteoprogenitor cells (49–53). There are perhaps several reasons for these discrepant findings. First, as noted above, estrogen deficiency, like aging, is associated with inflammation (47), but this proinflammatory state secondary to estrogen deficiency may not necessarily reflect a SASP. Second, several of the positive studies used commercial polyclonal antibodies against mouse *p16^Ink4a^* that have not been well validated and may lack appropriate specificity. Third, these studies generally relied on SA-β-gal staining to assess senescence, and none used more specific assays (e.g., SADS or TAFs) to definitively establish cell senescence following estrogen deficiency. Nonetheless, it is important to note that our study (48) as well as others (49–53) have examined estrogen deficiency–related bone loss in relatively young (4- to 6-month-old) mice. Thus, it is certainly possible that there is an interaction between long-term estrogen deficiency and aging effects on bone that warrants further investigation.

### Diabetes mellitus.

There is increasing evidence that cellular senescence contributes to the pathogenesis of both the insulin resistance and β cell failure in type 2 diabetes mellitus (reviewed in ref. 12). In addition, recent studies also demonstrate that cellular senescence may contribute to diabetic skeletal fragility (54, 55), which is related not to reductions in bone mass, but rather to abnormal bone “quality” (56–58). Factors that contribute to the impaired bone quality in diabetes include increased cortical porosity and impaired bone material properties related, at least in part, to the accumulation of advanced glycation end products (AGEs) (59, 60). In addition to directly damaging bone, AGEs bind the receptor for AGEs (RAGE) to activate signal transduction in multiple cell types and tissues, and over prolonged periods they drive inflammation and contribute to diabetic complications (61, 62).

To evaluate the role of cellular senescence in diabetic bone disease, Eckhardt et al. (54) first demonstrated in a high-fat diet/single-dose streptozotocin mouse model that these obese young adult mice did, indeed, develop postpubertal type 2 diabetes (T2D) and also had skeletal changes similar to those observed in humans with the disease, including impaired bone material properties and reduced bone formation rates. Concomitant with this, *p16^Ink4a^* and *p21^Cip1^* mRNA levels were higher in osteocyte-enriched bone samples of T2D as compared with control mice, along with an increase in senescent osteocytes (SADS^+^ and TAF^+^). In addition, these senescent osteocytes developed a unique proinflammatory SASP composed predominantly of upregulated levels of MMPs and NF-κB. These studies thus implicate cellular senescence in the skeletal changes associated with T2D, and studies are ongoing to test whether clearance of senescent cells ameliorates the skeletal fragility in this disease, as has been shown for age-related bone loss.

Similar findings have recently been reported by Gong et al. (55) in a mouse model of type 1 diabetes (T1D). These investigators found increased protein markers of cellular senescence (γ-H2AX, p16, p21, and p53) in femurs of diabetic mice. Interestingly, the increase in these markers could be prevented by the administration of melatonin, which also ameliorated the bone loss in the T1D mice. Thus, although the data are currently limited, there is evidence supporting a role for cellular senescence in mediating skeletal fragility in diabetes mellitus.

### Radiation and chemotherapy.

Radiation therapy is very effective in killing cancer cells but also has adverse bystander effects on bone, which include bone loss as well as osteoradionecrosis (63). In vitro, radiation induces cellular senescence by increasing oxidative stress, DNA damage, and chromatin disruption (18). In vivo, focal irradiation (24 Gy) of the femoral metaphysis in mice resulted in bone loss along with an increase in senescent (TAF^+^) osteocytes, bone lining cells, and osteoblasts at 42 days after radiation (64), consistent with the induction of cellular senescence. This was accompanied by increased *p21^Cip1^* and, to a lesser extent, *p16^Ink4a^* expression as well as increases in mRNA levels of 18 of 46 SASP genes analyzed (64). Moreover, treatment of irradiated mice with the senolytic cocktail of D+Q prevented radiation-induced bone loss as well as reduced senescent osteocytes and senescence/SASP markers in bone.

In addition to radiation, chemotherapy is also associated with the induction of cellular senescence in various tissues, including bone (65). Thus, mice treated with doxorubicin had increased expression of *p16^Ink4a^*, *p21^Cip1^*, and SASP genes in bone marrow mesenchymal cells (66). In addition, treatment with AP20187 was able to prevent doxorubicin-induced bone loss in young adult *INK-ATTAC* mice. Consistent with effects observed in aged *INK-ATTAC* mice, AP20187 treatment was also associated with a reduction in osteoclast numbers and increased bone formation rates in comparison with control mice treated with doxorubicin alone (66). Thus, both radiation- and chemotherapy-induced bone loss are associated with increased cellular senescence that can be ameliorated by a reduction in senescent cell burden.

## Potential physiological roles of cellular senescence in the skeleton

### Growth.

There is increasing evidence that cellular senescence, while clearly detrimental with aging and the other skeletal conditions reviewed above, may also play physiological roles. Thus, developmentally programmed cell senescence occurs during mammalian embryonic development, including the mesonephros and the endolymphatic sac of the inner ear (67), as well as the apical ectodermal ridge and the neural roof plate (68). In addition, cellular senescence is associated with the cessation of linear growth during puberty. Indeed, mesenchymal stem-progenitor cells in the primary spongiosa of the long bones in mice, which are highly proliferative during early puberty, undergo programmed senescence at late puberty. This is driven by an epigenetic mechanism whereby the enhancer of zeste homolog 2 (EZH2), the histone methyltransferase of polycomb repressive complex 2 that catalyzes histone H3 lysine 27 trimethylation (H3K27me3), maintains the self-renewal and proliferative capacity of primary spongiosa cells early in puberty, but loss of EZH2-H3K27me3 during late puberty leads to cell senescence and cessation of longitudinal growth (69).

Another aspect of skeletal development that is regulated by cellular senescence is the growth inhibition and osteoporosis induced by exogenous glucocorticoid therapy. Thus, Liu et al. (70) demonstrated that glucocorticoid treatment of young (3-week-old) mice induced vascular endothelial cell senescence in the metaphyses of long bones, which was driven by reduced production of angiogenin (a ribonuclease with proangiogenic activity) by osteoclasts. These data reveal a novel link between osteoclasts and their regulation of endothelial cell senescence as well as underlying mechanisms for glucocorticoid effects on the growing skeleton. However, whether a similar mechanism is involved in glucocorticoid-mediated osteoporosis in adult animals or humans requires further study.

### Fracture and tissue repair.

Another physiological role of senescent cells may be in tissue repair (71), although the beneficial versus detrimental effects of cellular senescence in this context remain incompletely understood and could be tissue specific. For example, following a skin wound, senescent fibroblasts and endothelial cells appear transiently in response to the injury and accelerate healing through the secretion of platelet-derived growth factor AA (PDGF-AA) (72). As such, it has been proposed that although senescence likely evolved as an anticancer mechanism, whereby cells that had accumulated oncogenic insults were redirected toward a senescent, growth-arrested phenotype rather than uncontrolled proliferation and cancer (8), another physiological role of senescent cells may be in the response to tissue injury, specifically by secreting cytokines and growth factors that attract immune cells to the site of injury and also activate repair mechanisms (71). In this formulation, once tissue repair is completed, the immune cells then clear the senescent cells in order to prevent the adverse consequences of a chronically increased senescent cell burden, and as noted earlier, it is likely that this immune clearance of senescent cells is impaired with aging, allowing them to accumulate (71).

In recent studies, we found that, similarly to skin injury, fracture in young adult mice is also associated with the transient accumulation of senescent (SADS^+^ and TAF^+^) cells along with increased expression of *p16^Ink4a^* and *p21^Cip1^*, as well as SASP markers, in the fracture callus (73). However, in contrast to the skin, clearance of senescent cells with D+Q did not impair but rather significantly accelerated the time course of fracture healing. Thus, it appears that the beneficial versus detrimental effects of senescent cells on injury repair may vary across tissues.

## Translational opportunities and challenges

In addition to D+Q (18), a number of other senolytics and senomorphics are in various stages of preclinical and early clinical development (reviewed in ref. 74). Indeed, initial proof-of-concept studies in humans have already been initiated for a number of diseases, including age-related bone loss (ClinicalTrials.gov NCT04313634). Clearly, as these trials progress, it will be important to define both the benefits, including the attractive possibility of treating multiple age-related comorbidities (e.g., osteoporosis, frailty, diabetes, cardiovascular disease, dementia) with a single or limited combination of senotherapeutics, and the potential risks of targeting senescent cells. The latter include concerns that preventing cells from becoming senescent may increase tumorigenesis because, as noted earlier, senescence may represent an anticancer mechanism (8). However, an important feature of senolytic drugs is that they appear to be effective when given intermittently (e.g., once a month, at least in mice; ref. 42), thereby decreasing the potential for off-target effects. In addition, these drugs will intermittently reduce the burden of senescent cells rather than prevent them from forming in the first place. Senescent cells may also enhance tumorigenesis through the proinflammatory SASP (8), and reducing the numbers of these cells and/or their SASP may, in fact, lead to reduced cancer development. Furthermore, senescent cells carrying oncogenic mutations may be eliminated by senolytics, several of which, including dasatinib, are currently used or being studied for preventing or treating cancers. Nonetheless, the potential safety concerns regarding senolytic therapies, including possible off-target effects beyond tumorigenesis, need to be carefully evaluated in the ongoing clinical trials. This issue applies also to senomorphics, with which, for example, the increased risk of viral infections and herpes zoster reactivation would need to be balanced against the possible benefits of suppression of the SASP by senescent cells (75).

## Summary and conclusions

There is now substantial evidence, primarily from animal models, that cellular senescence plays a key role in mediating age-related bone loss as well as bone fragility associated with a number of other conditions, including diabetes mellitus, radiation, and chemotherapy. Moreover, targeting senescent cells prevents bone loss associated with these conditions, at least in mice. As such, despite the availability of a number of therapeutic options specifically for treating osteoporosis, the prospect of placing osteoporosis treatment in the context of treating multiple other aging conditions offers perhaps renewed hope that we can bridge the growing gap in osteoporosis treatment (7). This approach does not involve developing new drugs for osteoporosis, but rather involves developing drugs that target a fundamental aging mechanism across tissues, thereby greatly amplifying the benefits versus the risks of these drugs.

## Author contributions

SK prepared an initial draft of the manuscript, which was reviewed and edited by JNF and DGM.

## Figures and Tables

**Table 1:**
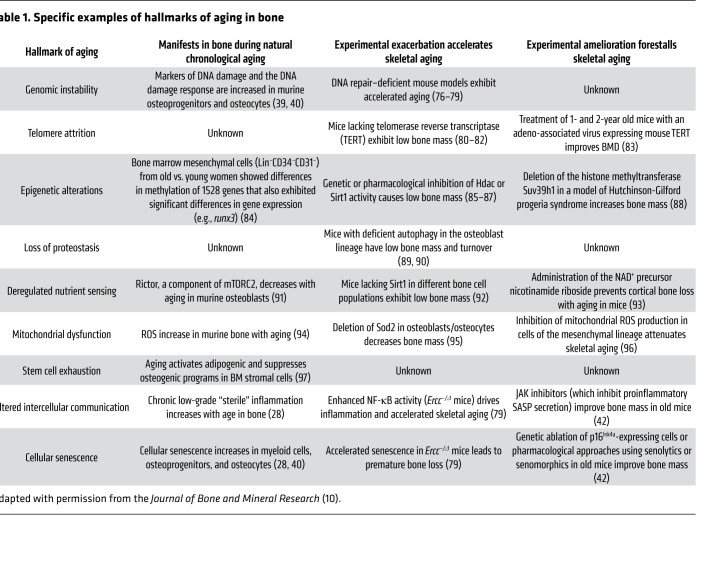
Specific examples of hallmarks of aging in bone

## Version 1 02/01/2022

Electronic publication
